# Supplementary material for: High-Sensitivity Magnetic Levitation Reveals Intrinsic Protein Corona Heterogeneity on Identical Nanoparticles
Source: bioRxiv. 2026 May 27:2026.05.23.727410. Preprint. [Version 1] doi: 10.64898/2026.05.23.727410 (PMC13232164; doi:10.64898/2026.05.23.727410)
Supplement: Supplement 1 [file NIHPP2026.05.23.727410v1-supplement-1.pdf]

bioRxiv preprint doi: https://doi.org/10.64898/2026.05.23.727410; this version posted May 27, 2026. The copyright holder for this preprint (which was not certified by peer review) is the author/funder, who has granted bioRxiv a license to display the preprint in perpetuity. It is made available under a CC-BY 4.0 International license.

High-Sensitivity Magnetic Levitation Reveals Intrinsic Protein Corona Heterogeneity on Identical Nanoparticles

Samantha Velazquez[1], William Thompson[2], Ali Akbar Ashkarran[2,3*]

[1]Department of Biology, University of Colorado Colorado Springs, Colorado Springs, CO, USA

[2]Department of Physics and Energy Science, University of Colorado Colorado Springs, Colorado Springs, CO, USA

[3]BioFrontiers Center, University of Colorado Colorado Springs, Colorado Springs, CO, USA

***Corresponding author:** (AAA) email: (aashkarr@uccs.edu)

bioRxiv preprint doi: https://doi.org/10.64898/2026.05.23.727410; this version posted May 27, 2026. The copyright holder for this preprint (which was not certified by peer review) is the author/funder, who has granted bioRxiv a license to display the preprint in perpetuity. It is made available under a CC-BY 4.0 International license.

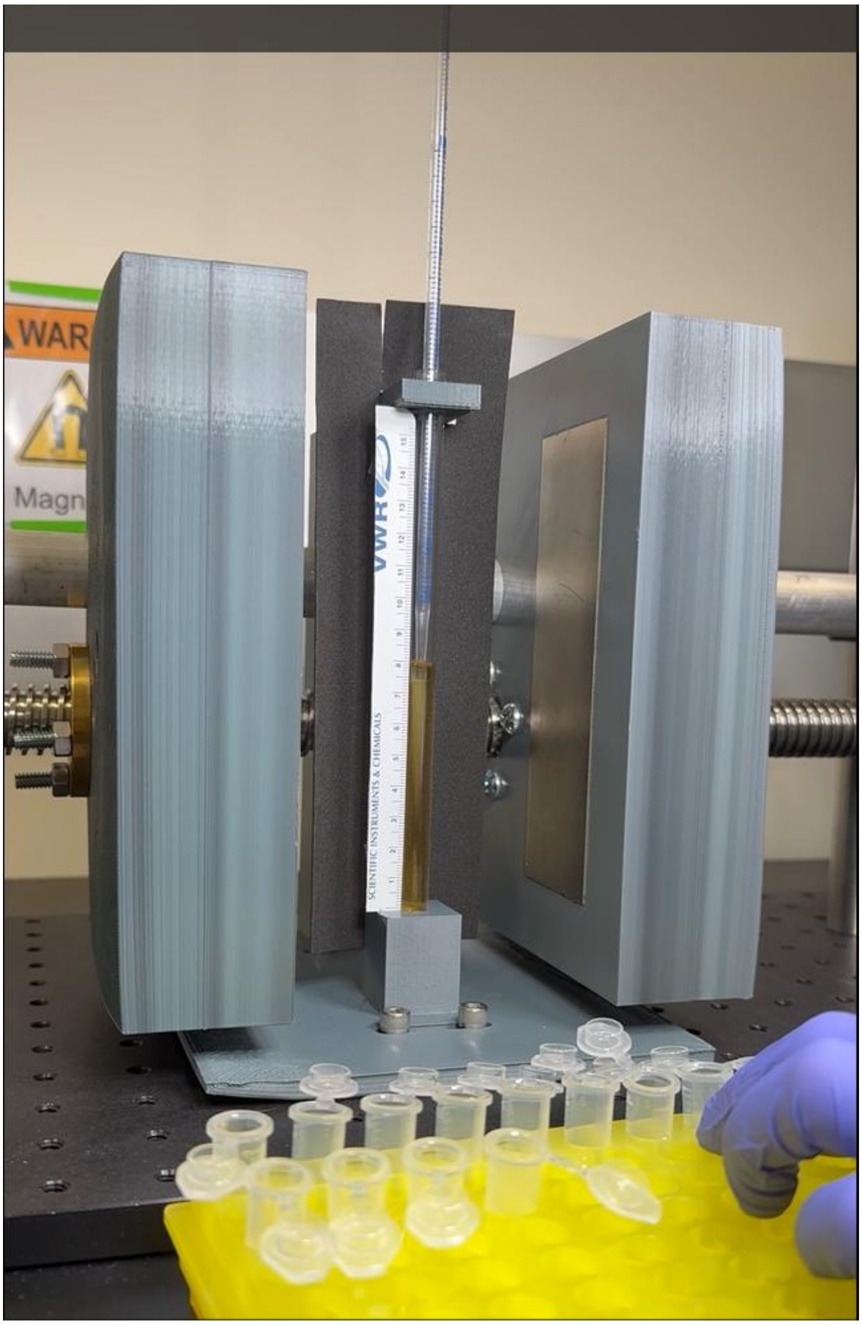

Figure S2: Photograph showing the extraction of different fractions from top to bottom of the high-sensitivity MagLev system using a micropipette attached to a 10 ml glass Pasteur pipette, with fractions collected at 1 cm intervals along the MagLev column and transferred to low binding Eppendorf tubes.

bioRxiv preprint doi: https://doi.org/10.64898/2026.05.23.727410; this version posted May 27, 2026. The copyright holder for this preprint (which was not certified by peer review) is the author/funder, who has granted bioRxiv a license to display the preprint in perpetuity. It is made available under a CC-BY 4.0 International license.

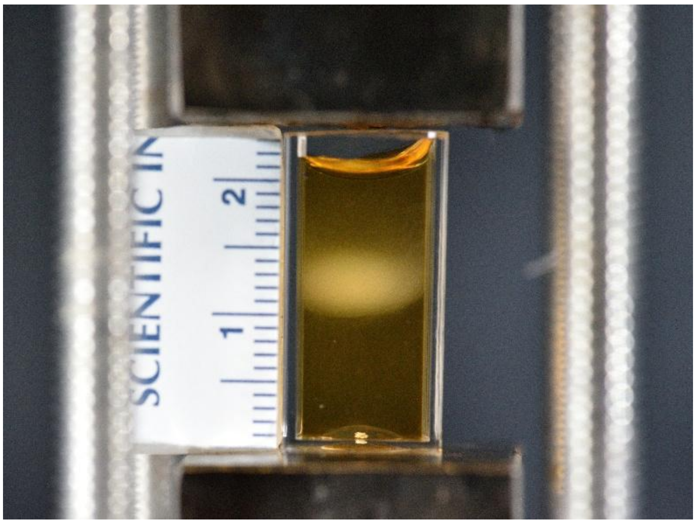

Figure S2: Levitation image of the PC-coated NPs in 0.125 mg/ml SPIONs in a standard MagLev system.

bioRxiv preprint doi: https://doi.org/10.64898/2026.05.23.727410; this version posted May 27, 2026. The copyright holder for this preprint (which was not certified by peer review) is the author/funder, who has granted bioRxiv a license to display the preprint in perpetuity. It is made available under a CC-BY 4.0 International license.

Table S1: Representative GO/pathway-related protein themes identified across the changing-protein dataset, with example proteins and corresponding biological interpretation.

Theme	Representative proteins	# of proteins	Interpretation
Keratin / structural / membrane-associated biology	KRT9, KRT14, KRT1, STOM	4	Indicates strong representation of structural and membrane-associated protein classes among the most dynamic fraction-dependent proteins.
Cytoskeleton / cell architecture / mechanostructure	TLN1, TUBB1, CORO1C	3	Supports fraction-dependent redistribution of cytoskeletal organization and cell-architecture related proteins.
Protein folding / chaperone-linked biology	CCT3	1	Suggests that protein-folding and proteostasis-related functions contribute to the strongest abundance changes across the column.
Metabolic / enzymatic proteins	PGK1, ADH4	2	Shows that metabolic enzymes are part of the strongest fraction-dependent protein shifts.
Plasma / extracellular-associated proteins	PROS1	1	Indicates that extracellular or plasma-associated signatures remain represented among the changing proteins.
Immune / Ig-like related proteins	Ig-like domain-containin	1	Suggests contribution of immune-related or immunoglobulin-like protein classes to the changing-protein set.

Analysis of the most dynamic proteins across the high-sensitivity MagLev fractions indicates prominent representation of structural, cytoskeletal, membrane-associated, metabolic, and selected extracellular protein classes, rather than a purely plasma- or immune-dominated signature alone. In particular, keratin- and membrane-associated proteins such as KRT9, KRT14, KRT1, and STOM, together with cytoskeletal and cell-architecture proteins such as TLN1, TUBB1, and CORO1C, represent the largest component of the top-changing set. This

bioRxiv preprint doi: https://doi.org/10.64898/2026.05.23.727410; this version posted May 27, 2026. The copyright holder for this preprint (which was not certified by peer review) is the author/funder, who has granted bioRxiv a license to display the preprint in perpetuity. It is made available under a CC-BY 4.0 International license.

suggests that the strongest fraction-dependent differences are related prominently to structural organization and protein assemblies associated with membrane or cytoskeletal states. Moreover, metabolic enzymes such as PGK1 and ADH4, the chaperone-linked protein CCT3, the plasma-associated protein PROS1, and an Ig-like domain-containing protein indicate that the fractionation also captures contributions from metabolism, proteostasis, extracellular biology, and immune-related signatures. The findings reveal that the high-sensitivity MagLev fractions differ not only in protein number and overlap, but also in protein class, with the top-changing proteins emphasizing structural and cytoskeletal remodeling alongside metabolic and extracellular-associated components.
